# Problem-oriented documentation: design and widespread adoption of a novel toolkit in a commercial electronic health record

**DOI:** 10.1093/jamiaopen/ooad005

**Published:** 2023-02-03

**Authors:** Richard L Altman, Chen-Tan Lin, Mark Earnest

**Affiliations:** Division of General Internal Medicine, Department of Medicine, University of Colorado Anschutz Medical Campus, Aurora, CO, USA; Division of General Internal Medicine, Department of Medicine, University of Colorado Anschutz Medical Campus, Aurora, CO, USA; Division of General Internal Medicine, Department of Medicine, University of Colorado Anschutz Medical Campus, Aurora, CO, USA

**Keywords:** medical records, problem oriented, user-computer interface, electronic health records, ambulatory care

## Abstract

**Background:**

Problem-oriented documentation is an accepted method of note construction which facilitates clinical thought processes. However, problem-oriented documentation is challenging to put into practice using commercially available electronic health record (EHR) systems.

**Objective:**

Our goal was to create, iterate, and distribute a problem-oriented documentation toolkit within a commercial EHR that maximally supported clinicians’ thinking, was intuitive to use, and produced clear documentation.

**Materials and Methods:**

We used an iterative design process that stressed visual simplicity, data integration, a predictable interface, data reuse, and clinician efficiency. Creation of the problem-oriented documentation toolkit required the use of EHR-provided tools and custom programming.

**Results:**

We developed a problem-oriented documentation interface with a 3-column view showing (1) a list of visit diagnoses, (2) the current overview and assessment and plan for a selected diagnosis, and (3) a list of medications, labs, data, and orders relevant to that diagnosis. We also created a series of macros to bring information collected through the interface into clinicians’ notes. This toolkit was put into a live environment in February 2019. Over the first 9 months, the custom problem-oriented documentation toolkit was used in a total of 8385 discrete visits by 28 clinicians in 13 ambulatory departments. After 9 months, the go-live education and EHR optimization teams in our health system began promoting the toolkit to new and existing users of our EHR resulting in a significantly increased uptake by outpatient clinicians. In April 2022 alone, the toolkit was used in more than 92 000 ambulatory visits by 894 users in 271 departments across our health system.

**Conclusions:**

As a health-system client of a commercial EHR, we developed and deployed a revised problem-oriented documentation toolkit that is used by clinicians more than 92 000 times a month. Key success elements include an emphasis on usability and an effective training effort.

## INTRODUCTION

### Background and significance

The idea of the problem-oriented medical record (POMR) was first described in landmark papers by Dr. Lawrence Weed in 1964 and 1968.^1^^,^^2^ He illustrated the need for a more “organized approach to the medical record.”^1^ At that time, the progress note addressed patients’ medical issues in bulk, muddling the course of individual conditions and perhaps causing inattention to critical concerns.^1^ Dr. Weed’s concept involved the creation of a note within a note, documenting each problem with its own contained subjective, objective, and assessment and plan (SOAP) section.

The POMR and SOAP concepts were adopted widely by the medical community including physicians, pharmacists, and nurses^3^ while a few dissenters advocated for more incremental reforms.^4^ By 1973, 73% of medical schools in the United States were teaching some form of the POMR to their students.^5^ Internationally, the POMR generated significant interest as well.^5–12^ Renewed attention to the POMR came to the United States in January 2021 as documentation guideline changes adopted by The Center for Medicare and Medicaid Services reduced the need for a specific number of physical exam findings and review of systems items, instead utilizing problem-focused medical complexity to demonstrate visit value.^13^^,^^14^ This new framework was compatible with a problem-oriented format in which to record relevant data, physical findings, and assessment and plans (A/P).

While the merits and deficits of the POMR have been debated,^3^^,^^15–20^ it remains an important concept that is supported by large electronic health record (EHR) systems.^21^^,^^22^ In the EHR used at our organization, the tools and data structure to support a POMR exist but in the experience of the authors, are infrequently used. Previous studies have observed the same and theorized that specific EHR limitations such as a cumbersome workflow and the number of steps needed to document in this format have limited its adoption.^15^^,^^21^ More general research has identified success factors for a POMR interface including context-sensitive data views, unification of documentation and data, easy access to problem-based data, efficiency, and ease of use.^23^^,^^24^ We theorized that the lack of POMR use was the result of an inefficient interface rather than a lack of enthusiasm for problem-oriented documentation and thus built a new interface over the existing architecture. We outline the design, implementation, and successful adoption of an alternate problem-oriented documentation toolkit within a commercial EHR. Our goal was to improve the ease of problem-oriented documentation and facilitate problem-focused clinical thinking.

## METHODS

### Setting

UCHealth is a Colorado-based 13-hospital medical system with nearly 4.5 million ambulatory visits a year. UCHealth uses a single instance of Epic EHR (Epic Systems, Verona, WI), which is shared across 18 hospitals and more than 600 ambulatory clinics.

As new hospitals have joined our system, we have deployed go-live teams to educate and train new users in our EHR. Additionally, UCHealth has multiple EHR optimization teams comprised of clinician informaticists, project managers, nurses, analysts, and trainers who perform single-clinic, intensive interventions focused on reducing clinician burnout through EHR and workflow efficiency.^25^^,^^26^

### Tools

The EHR provides a set of tools which allow modification of the user interface by the health-system client. We used these tools, based on “Physician Builder” training by the EHR vendor, along with additional programming tools using Caché^®^ (InterSystems Corporation, Cambridge, MA) to develop our interface.

### EHR-provided interface

**Figure 1.**
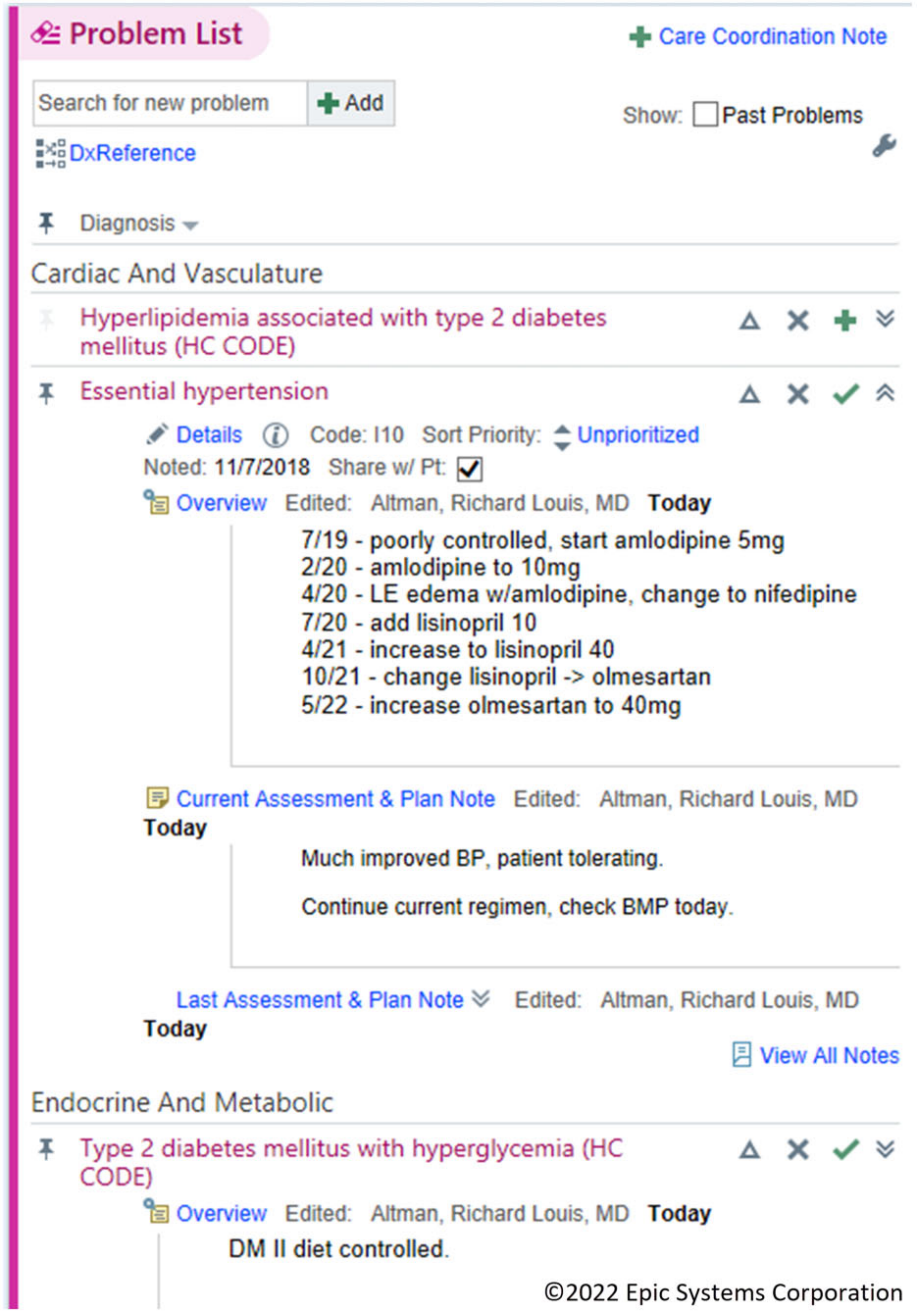
EHR native problem-oriented charting.

The EHR-provided interface for problem-oriented documentation is accessed through a patient’s problem list (Figure 1). In the problem list section, each diagnosis on the list has a space to document an overview note (a clinically important short summary of the diagnosis) and a visit-specific A/P note (plans for today). The overview and A/P documentation for these problem list diagnoses are saved for use at future visits. During each visit, clinicians can build a list of visit-specific diagnoses using both existing diagnoses from the problem list and acute diagnoses which are not on the problem list. Before the implementation of our toolkit, our primary care clinics used the native problem-oriented documentation tool in fewer than 10% of all visit A/Ps.

There are usability concerns with the existing interface as identified by the authors:

1. Writing an overview or A/P note requires hunting for the diagnoses on the problem list and clicking multiple times to open the specific documentation section.
2. Unlike a problem list diagnosis, there is no place to document the A/P for an acute diagnosis.
3. Viewing problem-specific data requires navigation to a new page.
4. There is no way to easily integrate documentation of acute diagnoses and problem list diagnoses in the visit note.

### Design principles

To improve the ease of use of problem-oriented documentation, we considered evidence-based design principles and user-centered concerns regarding EHRs. User interface design literature notes that spatial location is a fundamental component of memory, consistent spatial arrangement of interfaces helps facilitate ease of navigation, and that reducing interface confusion reduces cognitive work and enables users to be more creative.^27–30^ Clinician dissatisfaction with EHRs strongly relates to a lack of clinically integrated views to help with care and the sense of performing data entry without clinical benefit.^31^^,^^32^ Thus, in creating a custom problem-oriented documentation toolkit, our primary design goals stressed visual simplicity, data integration, a predictable interface, data reuse, and clinician efficiency.

To reduce task switching and support a problem-oriented process, we felt a single screen should contain the comprehensive information needed to make clinical decisions for a particular diagnosis. Information displayed in this screen should be shown in a predictable location and behave in an intuitive way requiring minimal education.^28^^,^^29^ To decrease duplicate work and task switching, historical information about problems should be easily accessible and integrated into the interface. Mouse clicks and scrolling were to remain at a minimum.

To improve the documentation burden, any information entered in our problem-oriented documentation interface should auto-generate a readable note which would be configurable to clinicians’ preferences.

We intended that any toolkit created would be a thin layer which maximized the use of EHR-provided tools to facilitate a transition to future versions of the EHR. To simplify customization, the toolkit would ideally provide analyst-level configuration options which would not require programmer support. The toolkit should also use the EHR’s native problem-oriented data structure to support the use of entered data in existing EHR interfaces and future upgrades.

### User-centered design

While this article describes an innovation which relied heavily on end-user feedback, we did not use a formal user-centered design protocol.

## RESULTS

### Toolkit creation

The initial creation took 1 physician, trained as a Physician Builder and Caché^®^ programmer, and working in the University of Colorado Division of General Internal Medicine, about 80 h of work. The first version of the toolkit was put into the live environment in February 2019.

### Toolkit workflow

**Figure 2.**
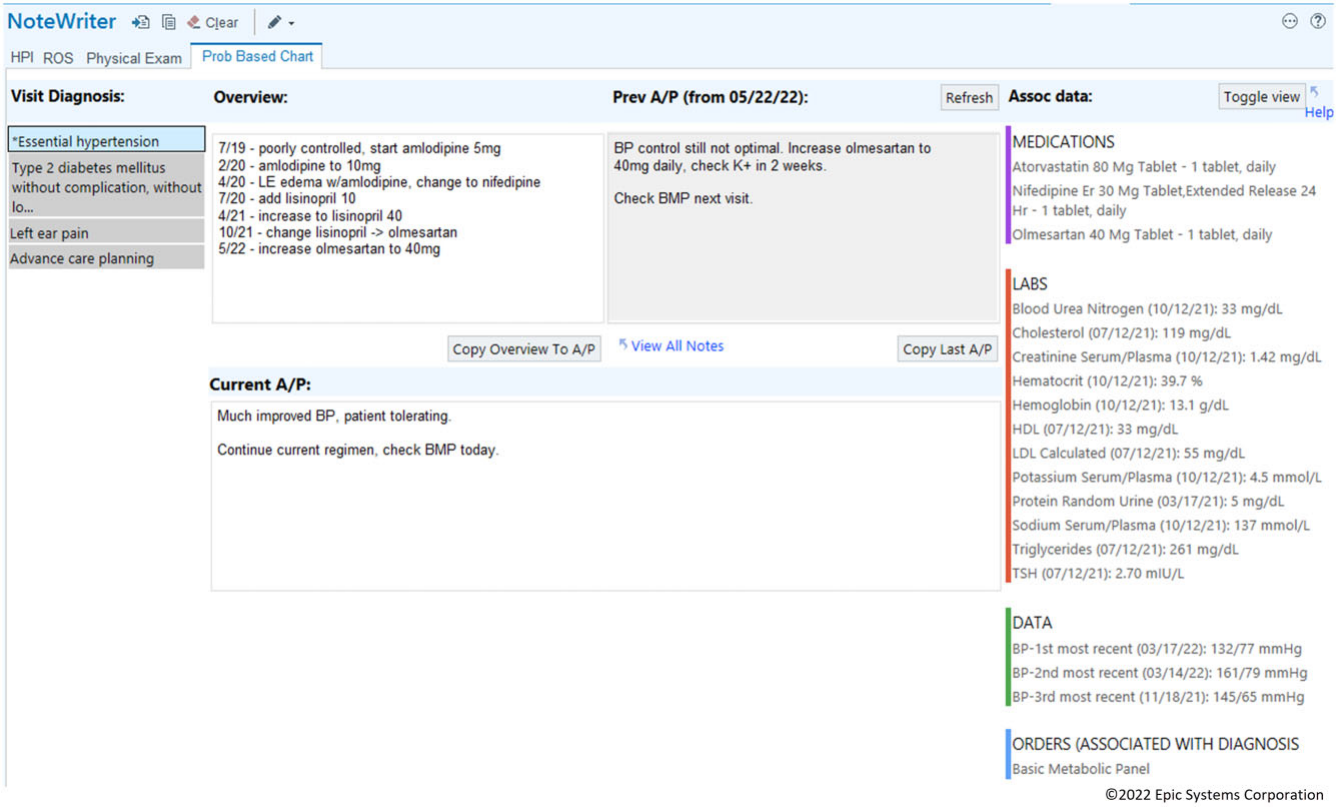
Custom problem-oriented charting interface.

During a visit, the interface (Epic SmartBlock SmartForm) is launched from the note editor and loads a view with 3 columns (Figure 2). The first column displays all diagnoses associated with the visit. These can be chronic diagnoses already on the problem list or acute diagnoses. The display dynamically mirrors the order in which these diagnoses appear on the EHR-managed visit diagnosis list. An asterisk (‘*’) designates the primary diagnosis for the visit. Clicking on a diagnosis in the first column populates its overview, previous A/P text, and current A/P text into stacked windows in the second column.

The top row of the second column starts with the diagnosis’ overview and the A/P from the most recent visit to the same department. The diagnosis’ A/P from the current visit is displayed in the bottom row of the second column. The diagnosis’ previous A/P from the most recent visit to the same department may be copied to the current A/P by pressing the “Copy Last A/P” button. A report displaying any previous A/P documentation can be viewed using the “View All Notes” hyperlink. A third column displays all diagnosis-associated medications, laboratory results, and other relevant data for this diagnosis as determined by expert clinical consensus gathered by our EHR optimization teams. This column also displays diagnosis-associated orders for the current visit.

A “Refresh” button updates any changes that may have been made to the data in other EHR interfaces. The “Toggle view” button selects among 4 different views of the data supporting user-level interface customization. A “Help” hyperlink leads to a webpage with a tip sheet for the interface.

### Note integration

**Figure 3.**
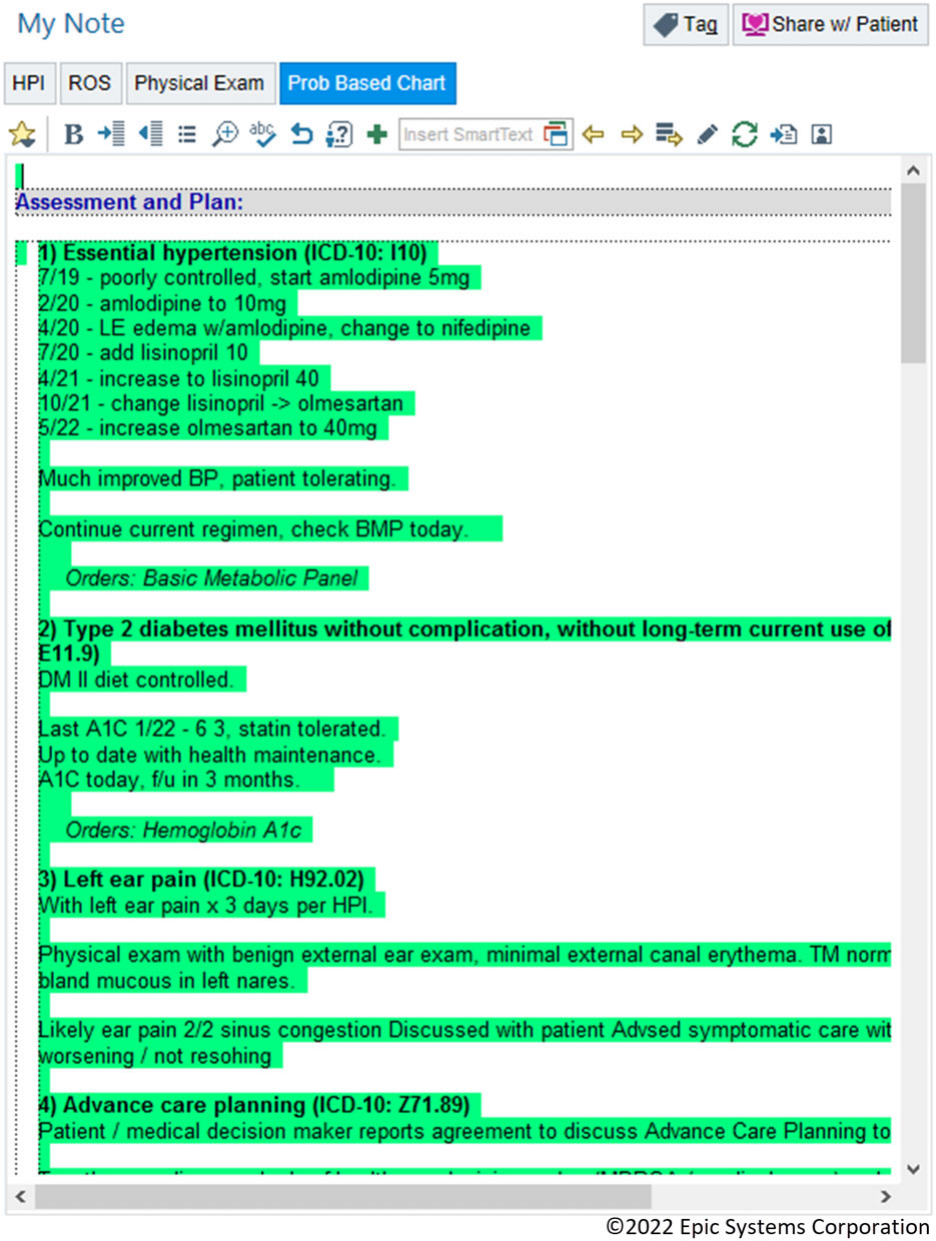
Import of problem-oriented charting data into note using macro.

Any information entered into the interface can be imported into the note by a refreshable macro (Epic SmartLink). Each diagnosis has a header which includes a number indicating its order in the visit diagnosis list as well as its ICD-10 code (Figure 3). Below this header appears the text of the overview, A/P, and associated orders. Refreshing the note updates changes made in the interface, among the orders, or to the visit diagnoses themselves. To facilitate individual clinician documentation styles, we created macros which allow users to specify which pieces of the problem-oriented data (overview, A/P, and orders) they wish to include in their note.

### Data integration

This new toolkit is tightly integrated with the EHR’s problem-oriented data structure. The overview and A/P data from the chronic problems on patients’ problem lists utilize the data elements of the native EHR such that information captured in our interface is immediately seen in the EHR native tool and vice versa.

### Adoption

The toolkit was initially soft-launched in the ambulatory setting with 2 physician-users in primary care (including author RLA) who actively tested for usability and errors. Changes based on real-world use resulted in minor interface changes and bug fixes. After a month of testing and iteration, we shared the toolkit with a wider group of ambulatory users who included medical directors of primary care clinics, physician informaticists, or physician problem-oriented documentation advocates. Feedback from this group also resulted in minor interface changes to the toolkit. Over 9 months of organic growth, the custom problem-oriented documentation toolkit was used in a total of 8385 discrete visits by 28 clinicians in 13 ambulatory departments.

Following this slow, natural adoption, 2 changes were made on a system level to further adoption. First, we integrated the toolkit in the default progress note used across the institution, placing it in the reach of all clinicians using our centralized note template. Second and more significantly, our physician-led go-live and EHR optimization teams began teaching the custom problem-oriented documentation toolkit as a part of their base curriculum. At new practice go-lives and within existing clinics, these teams gave at-the-elbow support to clinicians on our problem-oriented documentation interface, demonstrated how and why to use it, and made user-level changes to help facilitate customized workflows. The use of the toolkit was not mandated or forced in any way by these teams or the larger system.

With note integration and go-live and EHR optimization team adoption, use of our problem-oriented documentation toolkit significantly increased. Six months after note integration and go-live and EHR optimization team adoption, clinicians used the toolkit at more than 8000 discrete visits per month.

**Figure 4.**
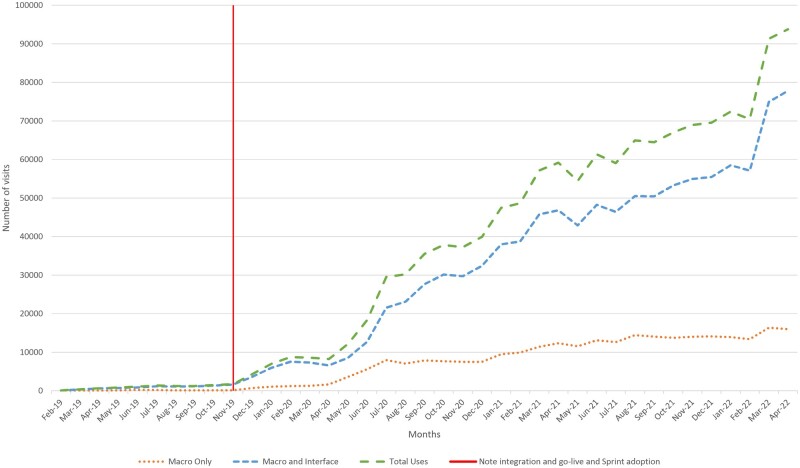
Custom problem-oriented charting toolkit use systemwide—2/2019 through 4/2022.

Since then, adoption has continued to trend up (Figure 4). In April 2022 alone, 894 clinicians used this problem-oriented documentation toolkit in 28% of all ambulatory visits across the health system (over 92 000 visits). During that month, it was used in 55% of primary care visits (more than 67 000 Internal Medicine, Family Practice, and Pediatrics visits), 42% of all gastroenterology visits (nearly 3000 visits), and over 32% of all OB/GYN/Women’s Health visits (over 8000 visits). During the entire study period, there were over 1 million individual uses of the toolkit; the toolkit is on trend for more than 1 million uses annually. Additionally, this toolkit has been successfully adopted at a second health system which reports more than 4000 daily uses.^33^

## DISCUSSION

### Summary

We demonstrate that it is possible to design a custom, problem-oriented documentation toolkit within a commercial EHR that can be widely adopted within 1 health system. A combination of factors contributed to this success: an iterative design, a simple and predictable interface, integrated clinical information, automatic note generation, and an effective education team. We have been able to lower the barrier to the intuitive POMR concept that Dr. Weed described by improving the workflow in our EHR. This toolkit shares design philosophy with the “Five Rights” framework of clinical decision support which advises delivering the right information, to the right person, in the right format, through the right channel at the right time in the workflow.^34^^,^^35^ Our implementation provides the right information (overview, previous A/P, current A/P, diagnosis-specific data), to the right person (the clinician writing a visit note), in the right format (a single-page display with consistent spatial arrangement and integrated data views), through the right channel (a user- launched interface), at the right time in the workflow (while editing the note).

Dr. Weed’s original concept of the POMR suggested that problem-focused data should be kept in the record alongside the documentation of the problem to facilitate organization and clinical thinking.^1^^,^^2^ Our toolkit does not include these data in the note itself but provides it visually to the user while clinical thinking is being applied to the problem, thus honoring the intent of Dr. Weed’s vision. The clinician is then free to include relevant data in their problem-focused documentation to explain their reasoning without furthering “note bloat.”

Previous studies have used existing EHR infrastructure to create problem-oriented concept maps to provide assistance and the reduce burden of clinical decision-making.^24^^,^^36^ Some implementations have used note templates, population-specific data collection, and a focus on increased utilization of native vendor tools to promote problem-oriented thinking.^37^^,^^38^ Similar solutions to ours have been proposed including access to previous notes on a particular problem, the association of relevant data, the inclusion of discrete objective and A/P sections, and an improved display.^39^ To our knowledge, this is the first problem-oriented documentation toolkit created using a commercial EHR and widely adopted at a system level.

### Strengths

The growth of problem-oriented documentation toolkit adoption benefited from buy-in and support from the EHR education teams who taught and promoted its use across the health system and were responsible for the large increases in its use. These teams were able to showcase the benefits of the new workflow in a unique and individual way which facilitated a shift change in the quantity and diversity of the user base. Active engagement from these educational influencers created a “push”; the features of the interface and macros themselves and the problem-oriented documentation concept engaged users, creating a “pull.”^40–43^ The dynamic tools and native problem-oriented data structure included in the EHR facilitated this toolkit. This project was made possible by the availability of a physician-programmer and a strong working relationship with system-level information technology partners.

### Limitations

The custom problem-oriented documentation toolkit is currently only validated for use in the outpatient setting of our system, potentially limiting its use to clinicians who work in that environment. As part of this study, we did not survey all Epic customers to investigate for similar tools. The toolkit was created at our institution and may be suited particularly well to our culture and workflow. We did not observe all users of the toolkit while using the interface; their experience may not reflect the intended use. Time and motion studies were not employed in studying this toolkit. Future work could track system-level overview and A/P use, better quantify the impact of go-live and EHR optimization teams, improve data views, and continue to explore the use of this toolkit at outside institutions to validate its design and workflow.

## CONCLUSION

As a health-system client of a commercial EHR, we developed and deployed a revised problem-oriented documentation toolkit that is voluntarily used by clinicians in 28% of all ambulatory visits. Key success elements include a user-centric, workflow-focused design which emphasizes technical usability coupled with an effective training effort. Our experience suggests that integration of our approach into the native EHR would promote the use of problem-oriented documentation.

## Data Availability

Data will be shared on request to the corresponding author with permission from UCHealth.
